# Analyzing Neoadjuvant Chemotherapy Effects in HER2-Low Breast Cancer: Real World Data

**DOI:** 10.7759/cureus.59652

**Published:** 2024-05-04

**Authors:** Marcelo Antonini, Andre Mattar, Fernanda G Richter, Marcellus N Ramos, Marina D Teixeira, Nathalia N Pantarotto, Nadia F Matta, Andressa G Amorim, Denise J Pinheiro, Reginaldo C Lopes

**Affiliations:** 1 Mastology Department, Hospital do Servidor Público Estadual – Francisco Morato de Oliveira, Sao Paulo, BRA; 2 Mastology Department, Hospital da Mulher, Sao Paulo, BRA

**Keywords:** her2-low breast cancer, neoadjuvant chemotherapy, receptor erbb-2, her2, breast cancer

## Abstract

Purpose: Neoadjuvant chemotherapy (NAC) can be used as upfront therapy in aggressive breast cancer (BC). human epidermal growth factor receptor 2 (HER2)-low BC, defined as tumors scoring +1 or +2 on immunohistochemistry without HER2 gene amplification by in situ hybridization, lacks information on real-world data (RWD) outcomes, especially in the NAC setting. This subgroup, which does not reach the HER2 positive criteria due to its lower receptor expression, represents a distinct clinical category potentially requiring tailored therapeutic approaches.

Study objective: The objective of this study is to characterize patients with BC with HER2-low status who received NAC in a Brazilian public reference center for female tumors and key outcomes such as pathological complete response (pCR), overall survival (OS), and metastasis-free survival (MFS).

Methods: A retrospective cohort study based on a large BC database from a reference cancer center in Brazil. Patients with BC that received NAC, diagnosed between 2011 and 2020, were included if they presented HER2-low status (defined as tumors scoring +1 or +2 on immunohistochemistry without HER2 gene amplification by in situ hybridization) and had complete data on outcomes. Clinical and demographic data were collected, such as age, menopausal status, Ki-67, hormone receptor expression and others. Key outcomes from the study comprised pCR (defined as ypT0/TIs/ypN0), overall survival, and metastasis-free survival (MFS). Survival analyses were conducted through the semiparametric Kaplan-Meier method to assess OS and MFS by pCR status, considering BC diagnosis as the index date.

Results: Overall, 297 patients were eligible and 141 were included in the study after matching the HER2-low definition. The pCR was seen in 18 out of 141 patients (12.7%). The median overall survival was 8.2 years, and the median MFS was 2.7 years. The OS of pCR was 83.4% and non-pCR was 58.1%; the DFS of pCR was 55.5% and non-pCR 40.6%.

Conclusion: This study gives updated insights on pCR, OS, and MFS in women with HER2-low BC exposed to NAC.

## Introduction

In the early 1990s, human epidermal growth factor receptor 2 (HER2) transitioned from a pre-clinical biomarker to a pivotal therapeutic target in HER2-positive breast cancer (HER2+ BC). Overexpression of HER2 is recognized as a poor prognostic factor in these cancers, making it an ideal target for treatment. Trastuzumab, a monoclonal antibody, was the first in a series of anti-HER2 therapies that revolutionized the management of HER2+ BC by significantly improving patient outcomes [1-3].

Following the introduction of trastuzumab, the development of additional anti-HER2 therapies has dramatically reshaped the treatment landscape for HER2+ BC. These advancements have not only shifted oncological practice but also fundamentally transformed the natural history of HER2+ BC, offering enhanced therapeutic options and improved prognoses for patients [4].

The evolving methodologies for characterizing HER2 status, particularly through the combined use of immunohistochemistry (IHC) and fluorescence in situ hybridization (FISH), have significantly refined our understanding of HER2 in breast cancer. These technologies have led to more precise assessments of HER2 expression levels, facilitating the categorization of tumors not only as HER2-positive but also into a distinct group known as HER2-low. Despite HER2-low expressing breast cancer, which includes tumors that do not meet the criteria for high HER2 expression but still show some level of HER2 protein, comprising about 50% of all breast cancer cases, the clinical significance of this category has been historically underexplored [5].

Recent studies and clinical trials specifically targeting the HER2-low group are proving pivotal. As international guidelines evolve to integrate new research findings, the recognition of HER2-low-expressing cancers has begun to shift treatment paradigms. These patients, previously considered ineligible for HER2-targeted therapies used in HER2-positive cases, are now being recognized as potential beneficiaries of newer, more finely-tuned HER2-targeted treatments. This shift not only broadens the therapeutic landscape but also underscores the importance of nuanced HER2 testing in personalizing treatment approaches, potentially improving outcomes for a considerable and previously underserved patient population [3-5].

Antibody drug conjugates (ADC) that bind to specific targets, such as HER2-expressing BC cells, have been developed as biomarker-directed proteins. When ADC comes in contact with a few HER2 surface proteins, it is incorporated and delivers an intra-cellular cytotoxic agent bound to the antibody. The first ADC used in HER2 tumors was TDM-1, which showed an improvement in disease-free survival in second-line treatment and also a possible treatment for pretreated HER2 advanced disease [6]. Another recent example of this HER2-directed antibody is trastuzumab deruxtecan, a compound that is conjugated with a topoisomerase I inhibitor and has been showing consistent results through many late-stage and highly treated BC populations. Trastuzumab deruxtecan has proved to be efficacious in HER2 super expressed tumors and also in low-expressing tumors and is being tested in earlier BC stages [7-10].

To date, there is still unapproved data on the use of ADC-targeting HER2-low patients eligible as a neoadjuvant treatment and a specific trial is addressing this scenario in DESTINY-Breast09 (NCT04784715). The early use of ADC for this HER2 profile might be a tendency given the multiple emerging evidence on the benefits of using ADC-targeting HER2 low agents in advanced stages [10]. In addition, patients eligible for neoadjuvant chemotherapy in the early-stage disease setting might face several additional challenges, such as the impact of high-quality surgery on outcomes, the role of pathological complete response as a surrogate endpoint, and other cancer-related characteristics of HER2 low profile that might still be not well characterized [11]. As new drug therapies are tested in clinical trials, which include a finite population with a strictly controlled environment, “day-to-day” evidence would be unprecedented [12] to inform the characteristics of HER2 low patients eligible for neoadjuvant chemotherapy [13]. Through a real-world data study, we aimed to characterize the demographics, clinical and survival outcomes of women with HER2-low expressing BC who are eligible for neoadjuvant chemotherapy (NAC).

## Materials and methods

Study design and data source

A retrospective cohort based on RWD (real world data or a database constructed not for the specific aim of this study but for monitoring the quality of health care assistance) at “Hospital do Servidor Público Estadual” (HSPE). As it is an analysis of the institution's database, the present study was approved by the Research Ethics Committee at HSPE (approval number CAAE 39097520.4.2001.0069) and exempted from the need for an informed consent form. International Society of Pharmacoepidemiology/International Society of Pharmacoeconomics and Outcomes Research (ISPE/ISPOR) guidelines [14] were used for the development of exploratory real-world study. Data from women diagnosed and treated in the aforementioned healthcare service from January 2011 to December 2020 were considered for the study.

Follow-up, inclusion and exclusion criteria

All participants were followed until the last consultation in the hospital (if alive) or death from diagnosis. Women with BC who were more than 18 years old and were diagnosed with invasive breast cancer treated with neoadjuvant chemotherapy were included in the study. Exclusion criteria comprised: women with inflammatory breast cancer, stage 4 at diagnosis, missing data, participation in clinical studies, and patients who did not receive adjuvant radiotherapy after conservative surgery and in cases of indicated mastectomy (tumor larger than five centimeters, more than one lymph node involved, compromised skin, compromised muscle).

The definition of HER2-low expression in breast cancer has been informed by earlier studies that categorized tumors with an immunohistochemistry (IHC) staining score of HER2 1+ or HER2 2+ combined with a negative fluorescence in situ hybridization (FISH) result as 'low-HER2 expressing cells.' This classification uses validated scoring systems to precisely determine the level of HER2 expression, which is crucial for identifying tumors with low but detectable HER2 protein levels. These scores typically reflect cases where HER2 protein is present in more than 0% but less than 10% of tumor cells (HER2 1+), or in cases of HER2 2+ where there is a moderate level of protein expression but no gene amplification as confirmed by FISH [9]. The definition of a positive hormone receptor was when there was more than 10% for the estrogen or progesterone receptor.

Outcomes and clinical data

As an RWD study with an exploratory focus, the primary outcome of the study was to characterize demographic and clinical information that could be associated with pCR, as well as the percentage of patients with HER2 low BC that achieve pCR. In addition, the study also assessed the overall survival (OS) and metastasis-free survival (MFS). OS was defined as the absence of death, confirmed through the presence of medical consultations in the database. MFS was defined as the absence of disease relapse (any site other than the primary BC) or absence of confirmed metastasis after diagnosis and use of neoadjuvant chemotherapy. We also collected information on the site of recurrence (local or systemic). Finally, according to specialty guidelines, pCR was defined as ypT0/Tis ypN0, assessed after surgical treatment. This classification underscores the absence of invasive cancer in the breast and lymph nodes, following the evaluation of the surgically resected tissue [15].

The clinical data collected for the study were age, weight, body mass index, and others. Data related to BC was defined as staging, type of breast cancer, ki-67, and cancer subtypes such as triple negative, luminal A and others. Treatment-related variables, such as the type of neoadjuvant chemotherapy, were also collected.

Analyses

Continuous variables were reported as means and standard deviations or medians and interquartile ranges, while categoric data was reported as the percentage and absolute count. We explored the relationship between clinical data and pCR by dividing patients into subgroups (pCR and non-pCR). To test if there was an association between clinical information and pCR, we used Fisher or Chi-square tests for categoric covariates and T-student for continuous variables when parametrically distributed data (eg.: age). OS and MFS were reported as Kaplan-Meier curves, and a log rank test was used to assess differences in pCR and non-pCR subgroups. Both OS and MFS curves considered five years as the cut-off for right censoring. All inferential analyses considered p-value <0.05 as the statistically significant cut-off.

Finally, using univariate logistic regression, we explored the relation of pCR with age, menopausal status, presence of progesterone and estrogen receptors, HER2-low status (if HER2 ++ or HER2 +/FISH negative), ki-67 and nuclear grade. This analysis was reported through a forest plot using odds ratio, 95% confidence interval, and p-value.

The sample for this study was based on the fact that the number of participants in the study should be representative of a population with HER2-low BC patients exposed to NAC. As this data is poorly reported in Brazil, we considered that in a population of women with BC who undergo surgery, on average, 28% of patients are exposed to NAC [16]. From that, we estimated that around 50% are HER2-low disease [3]. That is, considering the two previous estimates, the probability of finding a HER2 low BC in a group of patients eligible for NAC is 14% (28% x 50%). Considering a 90% power, 10% margin of error and 5% alpha, at least 47 women with HER2 low BC who underwent NAC would be required.

## Results

Sample characteristics and neoadjuvant chemotherapy in HER2-low BC

**Figure 1:**
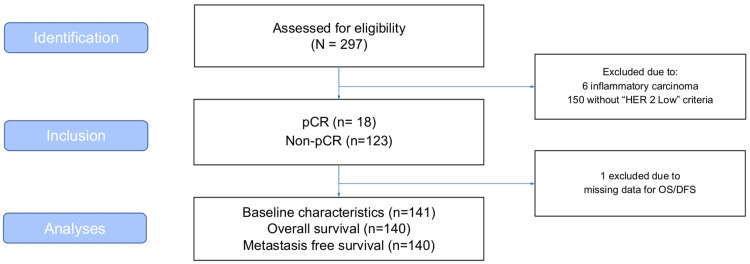
Sample characteristics and neoadjuvant chemotherapy in HER2-low BC BC: Breast cancer.

From 2011 to 2020, 297 patients were initially considered eligible for the study based on registries confirming surgery and use exposure to NAC. After a thorough review, six patients were excluded due to inflammatory carcinoma and 150 did not present HER2 low criteria. In the end, 141 patients were included for baseline analysis and 140 were included for OS and MFS analyses (one patient was excluded due to missing data) (Figure 1).

A total of 18 out of 141 (12.7%) patients with HER 2-low BC eligible for NAC presented pCR. At diagnosis, these women were 54 years old on average, with 27.5 kg/m^2^ (body mass index) and 13 (72%) in post-menopausal status. Most of the patients did not present a history of cancer in the family. The most common BC was ductal histologic type, with 11 (61.1%) of them presenting estrogen receptors and 10 (56,6%) presenting progesterone receptors. In this cohort, HER 2-low was typically HER2 +1 type 11 (61,1%) with an average ki-67 of 25%. The most common NAC was anthracycline, cyclophosphamide + taxane (AC+T) 17 (94,6%) followed by AC+T carboplatin 1 (5.6%) (Table 1).

**Table 1:** Baseline breast cancer patients characteristics with pCR and non-pCR pCR: Pathological complete response; BMI: Body mass index; A: Anthracycline; T: Taxane; C: Cyclophosphamide. Continuous variables: Student's t-test and Categorical variables: Chi-square and Fisher test.

Characteristic	pCR (n=18)	non-pCR (n=123)	p-value
Age, years; mean [sd]	54.6 [8.9]	54.7 [9.13]	0.965
Weight; mean [sd]	71.2 [16.4]	69.9 [14.8]	0.732
Height; mean [sd]	1.58 [0.06]	1.58 [0.06]	1
BMI; mean [sd]	28.3 [5.74]	27.9 [5.44]	0.773
Menopausal status, n (%)			
Pre-menopausal	5 (28)	37 (30.1)	0.783
Post-menopausal	13 (72)	86 (69.9)	
Family history of cancer, n (%)			
First-degree^α^	3 (16.6)	13 (10.6)	0.416
Second-degree^β^	3 (16.6)	16 (13)	
No family history, n (%)	7 (38.8)	66 (53.6)	
Tumor histologic type, n (%)			
ductal	18 (100)	117 (98.7)	0.783
lobular	0 (0)	6 (4.8)	
others	0 (0)	0 (0)	
Histological grade, n (%)			
1	6 (33.3)	22 (17.8)	0.298
2	7 (38.9)	63 (51.2)	
3	5 (27.8)	38 (30)	
Nuclear grade, n (%)			
1	6 (33.3)	22 (17.8)	0.298
2	7 (38.9)	63 (51.2)	
3	5 (27.8)	38 (30)	
Angiolymphatic invasion, n (%)			
Yes	1 (5.5)	15 (12.2)	0.562
No	17 (94.5)	108 (87.8)	
Lymphocyte infiltration, n (%)			
Yes	14 (77.8)	111 (90.2)	0.079
No	4 (22.2)	12 (9.8)	
Perineural invasion, n (%)			
Yes	1 (5.5)	10 (8.1)	0.816
No	17 (94.5)	113 (91.9)	
Estrogen receptor, n (%)			
positive	11 (61.1)	84 (68.3)	0.454
negative	7 (38.9)	39 (31.7)	
Progesterone receptor, n (%)			
positive	10 (55.6)	75 (60.9)	0.661
negative	8 (44.4)	48 (39.1)	
HER2 status, n (%)			
HER 2+ and FISH negative	7 (28.9)	47 (38.2)	0.955
HER 1+	11 (61.1)	76 (61.8)	
KI-67, %; mean [sd]	25 [21.2]	25 [22.7]	0.888
Clinical T, n (%)			
1	0 (0)	2 (1.6)	0.599
2	9 (50)	58 (47.2)	
3	8 (44)	42 (34.1)	
4	1 (5.6)	21 (17.1)	
Clinical N, n (%)			
0	5 (27.8)	37 (30)	0.881
1	6 (33.3)	46 (37.4)	
2 - 3	7 (39.2)	40 (32.6)	
Clinical TNM, n (%)			
T0N0	0 (0)	0 (0)	
TisN0	0 (0)	0 (0)	
T0N1	0 (0)	0 (0)	
T0N2	0 (0)	0 (0)	
T0N3	0 (0)	0 (0)	
T1N0	0 (0)	1 (0.8)	
T1N1	0 (0)	0 (0)	
T1N2	0 (0)	0 (0)	
T2N0	2 (11.1)	23 (18.7)	
T2N1	4 (22.2)	22 (17.9)	
T2N2	3 (16.7)	13 (10.6)	
T2N3	0 (0)	0 (0)	
T3N0	3 (16.7)	17 (13.8)	
T3N1	2 (11.1)	17 (13.8)	
T3N2	2 (11.1)	8 (6.5)	
T3N3	1 (5.56)	0 (0)	
T4N0	0 (0)	1 (0.8)	
T4N1	0 (0)	7 (5.6)	
T4N2	1 (5.56)	13 (10.6)	
T4N3	0 (0)	0 (0)	
Neoadjuvant chemotherapy, n (%)			
AC+T	17 (94.4)	120 (97.6)	0.252
AC+TCarboplatin	1 (5.6)	3 (2.4)	

**Figure 2:**
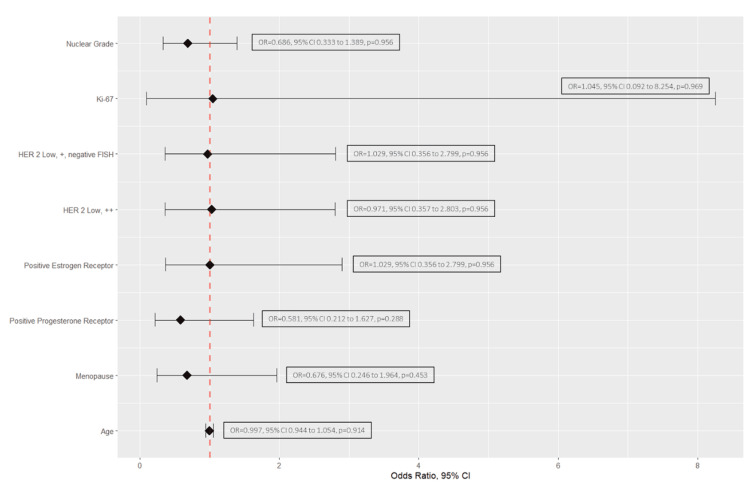
Forest plot of predictor factors of pCR in HER2-low patients. pCR: Pathological complete response.

The univariate analyses (Table 2) revealed that age, menopause status, positive estrogen and progesterone receptors, HER2-low definition (if HER2 ++ or HER2 +/FISH negative), ki-67, and nuclear grade were poor predictors for pCR (Figure 2).

**Table 2:** Univariate analyses of HER2-low dataset predicting pCR pCR: Pathological complete response.

Outcomes	pCR (n=18)	non-pCR (n=123)	p-value
Local Recurrence, n (%)	3 (16.7)	4 (3.2)	0.024
Axillary	0 (0)	1 (0.8)	
Breast	1 (5.56)	3 (2.4)	
Lymph node	0 (0)	1 (0.8)	
Skin	1 (5.56)	0 (0)	
Thoracic wall	1 (5.56)	0 (0)	
Systemic recurrence, n (%)	2 (11.1)	28 (22.7)	0.289
Bone	1 (5.56)	13 (10.6)	
Brain	0 (0)	6 (4.8)	
Liver	2 (11.1)	9 (7.3)	
Lung	2 (11.1)	14 (11.4)	
Lymph node	0 (0)	2 (1.6)	
Ovary	0 (0)	1 (0.8)	
Skin	1 (5.56)	1 (0.8)	
Deaths	3 (16.7)	41 (33.3)	0.141

Local or systemic recurrence, OS, MFS and univariate analyses

Overall, there were seven events of local recurrence (three pCR vs. four non-pCR, p=0.024) and 30 systemic recurrences (two pCR vs 28 non-pCR, p=0.289). The most common sites for systemic recurrence were markedly lung, bones, and liver (Table 3).

**Table 3:** Local recurrence and systemic recurrence (metastasis) of HER2-low patients

Variable	P	OR	95%CI lower	95%IC upper
Age	0.914	0.997	0.944	1.054
Menopausal status	0.453	0.676	0.246	1.964
Progesterone receptor	0.288	0.581	0.212	1.627
Estrogen receptor	0.991	1.005	0.369	2.901
HER2				
+2 with negative FISH	0.956	1.029	0.356	2.799
+1	0.956	0.971	0.357	2.803
Ki-67	0.969	1.045	0.092	8.254
Nuclear grade	0.297	0.686	0.333	1.389

**Figure 3:**
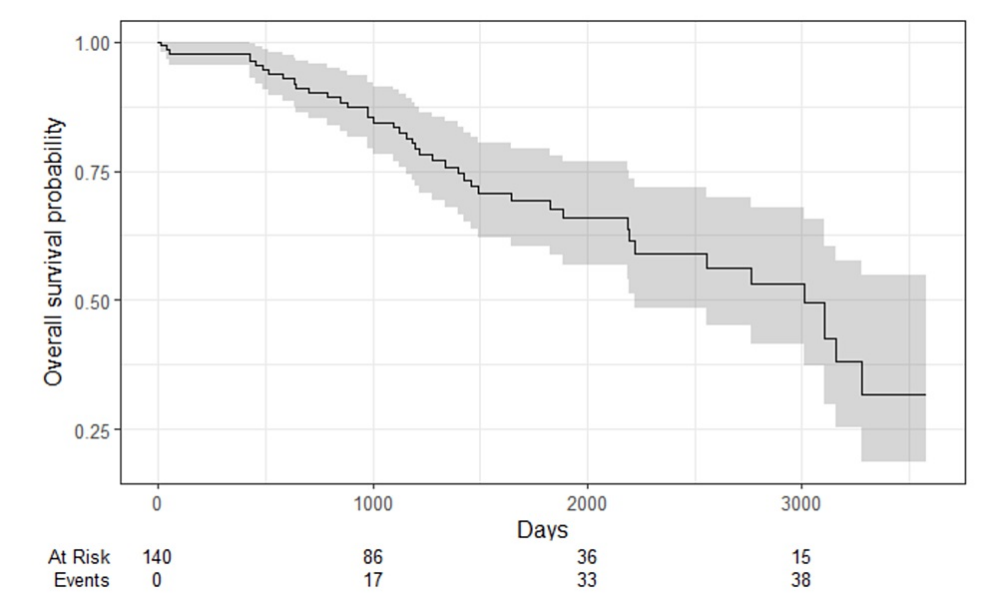
Overall survival of HER2-low patients and NAC NAC: Neoadjuvant chemotherapy.

**Figure 4:**
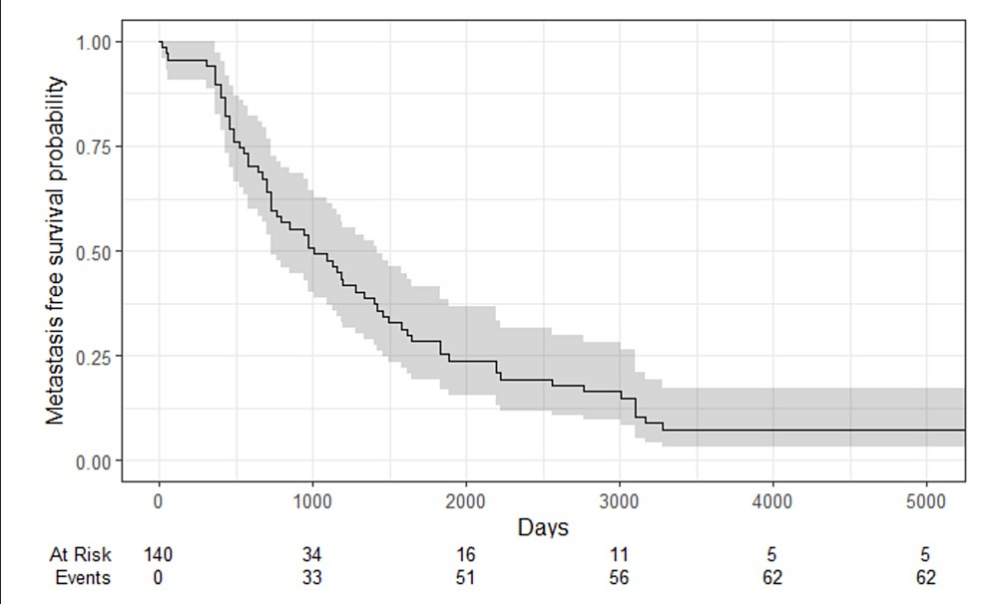
Metastasis-free survival of HER2-low patients and NAC NAC: Neoadjuvant chemotherapy.

The median OS was 8.2 years or 3000 days (Figure 3), while the median MFS was 2.7 years or 1000 days (Figure 4). The OS of pCR was 83.4%, and non-pCR was 58,1%; the DFS of pCR was 55.5% and non-pCR was 40.6%.

## Discussion

To our best knowledge, this is the first study that characterized patients with HER2 low BC who were eligible for NAC and provided an analysis of OS, MFS, and pCR patterns in a population that still is not well characterized. With the upcoming tendency to use ADC in HER2-low expressing BC, this study gives important real world driven insights [10].

Despite being close to 50% of all HER2 expressing BC, the HER2 low population was the subject of many criticisms and its characterization was not consensual by the scientific community for many years. One of the key findings from this study includes the characterization of HER2 low status, where it was noted a higher incidence of HER2+, compared to HER2++/FISH negative. Such finding is aligned with other ongoing studies on NAC-eligible BC which suggested a 70% HER2+ expression [17].

Another key finding of the study was the pCR rate (12%). One ongoing clinical trial about trastuzumab deruxtecan used as NAC in early BC revealed a close number (12%) of pCR in the entire intention to treat population. In that study, three out of 17 in the trastuzumab deruxtecan alone arm and one out of 16 in the trastuzumab deruxtecan plus endocrine therapy (12% considering three out of 33 patients). Though it is accepted that pCR is a relevant surrogate outcome in BC literature after NAC, the relation between pCR and hard outcomes is not completely clear. Interestingly, the KEYNOTE-522 trial (triple negative NAC eligible population) suggested a high pCR rate (around 60%) in patients receiving pembrolizumab [18]. This information might reinforce the association of HER2-expressing cells with poorer outcomes, likewise reported in other HER2-positive studies in women with BC that received trastuzumab as NAC (38% pCR rate) [19,20]. Other studies on the HER-positive population suggested higher rates of pCR when using pertuzumab added to trastuzumab emtasine, docetaxel, and carboplatin (55%) [21]. In other words, considering the existing evidence so far, ADC trials revealed discrete improvements in pCR in HER2 low population eligible for NAC. Our univariate analyses confirm that the presence of pCR was not predicted by common baseline characteristics used to eligibility for NAC, such as age, receptor status, ki-67, and menopausal status [22-23]. 

Improvements in OS and event-free survival, DFS, or MFS might be clinically even more challenging to interpret in HER2-low BC eligible for NAC. It is well-known that in previous studies of non-HER2 low BC (such as NOAH and KEYNOTE-522), the median OS was not achieved. The median survival and MFS were respectively characterized as 8.2 and 2.7 years in the present study, which is markedly below previous estimates on triple negative BC and HER2-positive populations eligible for NAC [19,24].

There was a significant difference between the number of local recurrences in the pCR and non-pCR groups, with a higher percentage in the pCR group. This must have been observed firstly due to the reduced number of patients with pCR and secondly due to the subtype of tumors, of these patients, who were HER-2 low patients with negative HR, who are considered TNBC, thus with a worse prognosis.

Several positive points and limitations may be found when dealing with real world data. First, assessing available database as source of real-world data is cheap compared to clinical trials or large prospective observational studies. In addition, it might be the only available resource to assess a population disease, likewise we did. However, the quality of the study and is highly dependent on the registries of the setting.

The main limitations of this study might be exemplified by the single-center data collection. Though the database available was not initially developed to perform RWD studies, local hospital managers aimed at monitoring the quality of care provided to BC women. Additionally, some of the parameters, such as the pCR rate and HER2+ status were similar to previous estimates, suggesting a logical association and the possibility to make the present research a benchmark for future studies conducted in other centers. Despite the limitations of a retrospective study with a small number of patients, the provocation for the need for larger studies of RWD in order to find answers to the behavior of these patients outside the RCT, even to be able to design a better RCT for the treatment of these HER-2-low patients with specific target therapies.

Through a study of real-world data, we were able, for the first time to our best knowledge, to characterize patients with low HER2 BC who were eligible for NAC and provide an analysis of patterns of OS, MFS and pCR in a population that has not yet is well characterized. This study is a landmark study because it characterized the NAC-eligible HER2-low BC population using real-world data sources. This research provides data on important demographics and clinical outcome information There is scope to characterize the HER2-low population in the NAC-eligible setting to evaluate whether ADCs focused on this BC subtype may be effective, as they have been shown to improve outcomes in advanced-stage disease.

## Conclusions

In conclusion, we did not find significant differences between HER-2-low patients with pCR and non-pCR. Ki67 and histological grade are markers for demonstrating low pCR rates. Patients with pCR have an overall survival of 8.2 years on average and a metastasis-free survival of 2.7 years. This retrospective single-center trial gives updated insights on pCR, OS, and MFS in women with HER 2-low BC exposed to NAC.

## References

[1] de Moura Leite L, Cesca MG, Tavares MC (2021). HER2-low status and response to neoadjuvant chemotherapy in HER2 negative early breast cancer. Breast Cancer Res Treat.

[2] 2] Miglietta F, Griguolo G, Bottosso M (2021). Evolution of HER2-low expression from primary to recurrent breast cancer. NPJ Breast Cancer.

[3] Marchiò C, Annaratone L, Marques A, Casorzo L, Berrino E, Sapino A (2021). Evolving concepts in HER2 evaluation in breast cancer: heterogeneity, HER2-low carcinomas and beyond. Semin Cancer Biol.

[4] Shirman Y, Lubovsky S, Shai A (2023). HER2-low breast cancer: current landscape and future prospects. Breast Cancer (Dove Med Press).

[5] Walko CM, West HJ (2019). Antibody drug conjugates for cancer treatment. JAMA Oncol.

[6] Verma S, Miles D, Gianni L (2012). Trastuzumab emtansine for HER2-positive advanced breast cancer. N Engl J Med.

[7] Diéras V, Miles D, Verma S (2017). Trastuzumab emtansine versus capecitabine plus lapatinib in patients with previously treated HER2-positive advanced breast cancer (EMILIA): a descriptive analysis of final overall survival results from a randomised, open-label, phase 3 trial. Lancet Oncol.

[8] Modi S, Saura C, Yamashita T (2020). Trastuzumab deruxtecan in previously treated HER2-positive breast cancer. N Engl J Med.

[9] Hurvitz SA, Hegg R, Chung WP (2023). Trastuzumab deruxtecan versus trastuzumab emtansine in patients with HER2-positive metastatic breast cancer: updated results from DESTINY-Breast03, a randomised, open-label, phase 3 trial. Lancet.

[10] Modi S, Jacot W, Yamashita T (2022). Trastuzumab deruxtecan in previously treated HER2-low advanced breast cancer. N Engl J Med.

[11] Basmadjian RB, Kong S, Boyne DJ (2022). Developing a prediction model for pathologic complete response following neoadjuvant chemotherapy in breast cancer: a comparison of model building approaches. JCO Clin Cancer Inform.

[12] Ferreira CG, Abadi MD, de Mendonça Batista P, Serra FB, Peixoto RB, Okumura LM, Cerqueira ER (2021). Demographic and clinical outcomes of Brazilian patients with stage III or IV non-small-cell lung cancer: real-world evidence study on the basis of deterministic linkage approach. JCO Glob Oncol.

[13] Venetis K, Crimini E, Sajjadi E (2022). HER2 low, ultra-low, and novel complementary biomarkers: expanding the spectrum of her2 positivity in breast cancer. Front Mol Biosci.

[14] Berger ML, Sox H, Willke RJ (2017). Good practices for real-world data studies of treatment and/or comparative effectiveness: Recommendations from the joint ISPOR-ISPE Special Task Force on real-world evidence in health care decision making. Value Health.

[15] Amin MB, Greene FL, Edge SB (2017). The eighth edition AJCC Cancer Staging Manual: continuing to build a bridge from a population-based to a more "personalized" approach to cancer staging. CA Cancer J Clin.

[16] Cammarota MC, Esteves BP, Daher JC (2013). Neoadjuvant chemotherapy and immediate breast reconstruction: a good option?. Revista Brasileira de Cirurgia Plástica.

[17] Hurvitz AS, Wang LS, Chan D (2021). TRIO-US B-12 TALENT: phase II neoadjuvant trial evaluating trastuzumab deruxtecan with or without anastrozole for HER2-low, HR+ early-stage breast cancer. J Clin Oncol.

[18] Lucas MW, Kelly CM (2022). Optimal choice of neoadjuvant chemotherapy for HER2-negative breast cancer: clinical insights. Cancer Manag Res.

[19] Laas E, Bresset A, Féron JG (2021). HER2-positive breast cancer patients with pre-treatment axillary involvement or postmenopausal status benefit from neoadjuvant rather than adjuvant chemotherapy plus trastuzumab regimens. Cancers (Basel).

[20] Gianni L, Eiermann W, Semiglazov V (2014). Neoadjuvant and adjuvant trastuzumab in patients with HER2-positive locally advanced breast cancer (NOAH): follow-up of a randomised controlled superiority trial with a parallel HER2-negative cohort. Lancet Oncol.

[21] Hurvitz SA, Martin M, Symmans WF (2018). Neoadjuvant trastuzumab, pertuzumab, and chemotherapy versus trastuzumab emtansine plus pertuzumab in patients with HER2-positive breast cancer (KRISTINE): a randomised, open-label, multicentre, phase 3 trial. Lancet Oncol.

[22] Vila J, Mittendorf EA, Farante G (2016). Nomograms for predicting axillary response to neoadjuvant chemotherapy in clinically node-positive patients with breast cancer. Ann Surg Oncol.

[23] Hwang HW, Jung H, Hyeon J (2019). A nomogram to predict pathologic complete response (pCR) and the value of tumor-infiltrating lymphocytes (TILs) for prediction of response to neoadjuvant chemotherapy (NAC) in breast cancer patients. Breast Cancer Res Treat.

[24] Schmid P, Cortes J, Pusztai L (2020). Pembrolizumab for early triple-negative breast cancer. N Engl J Med.

